# The Merit Release Birds: Buddhist ritual and implications in the H5N1 virus contamination cycle

**Published:** 2011-01-10

**Authors:** Ramona Alikiiteaga Gutiérrez, Philippe Buchy

**Affiliations:** 1 Institut Pasteur du Cambodge, Phnom Penh, Cambodia

## Background

The Highly Pathogenic Avian Influenza (HPAI) H5N1 virus has dramatically spread throughout Southeast Asia since its first detection in 1997. Merit Release Birds, such as the Eurasian-Tree sparrow, are believed to increase one’s positive karma when kissed and released during Buddhist rituals. Since these birds are often in close contact with both poultry and humans, we investigated their potential role in the spread of H5N1 virus in Cambodia, a Buddhist country were H5N1 virus is endemic.

## Methods

Specific Pathogen Free (SPF) chickens were exposed to Eurasian-Tree sparrows inoculated with HPAI H5N1 virus. In a second series of experiments, Eurasian-Tree sparrows were exposed to SPF ducks inoculated with HPAI H5N1 virus. Tracheal and fecal samples were collected daily from all animals. After 15 days, the surviving birds were euthanized and autopsied. Samples were tested for H5N1 virus by real-time RT-PCR and egg inoculation. All experiments were conducted under Biosafety level 3+ conditions.

## Results

When directly inoculated, Eurasian-Tree sparrows were susceptible to the H5N1 viral infection, with a fatality rate approaching 100% by 5 days post-inoculation (dpi). However, they did not contaminate the chickens maintained in the same isolator. SPF ducks were also highly sensitive to the HPAI infection, with a fatality rate of 80 to 90% within 8 dpi. Twenty percent of the naïve Eurasian-Tree sparrows which were in direct contact with the infected ducks in the isolator died from H5N1 infection. Large quantities of H5N1 virus were detected in the sparrows, particularly in their feathers.

## Conclusion

Our study indicates that under experimental conditions, Eurasian-Tree sparrows are susceptible to HPAI H5N1 infection, either by direct inoculation or by contact with infected poultry. Although the HPAI H5N1 virus was detected in sparrow trachea and faeces, we did not conclusively demonstrate a risk of poultry contamination by infected Sparrows. However, the presence of significant quantities of H5N1 virus on sparrow feathers would suggest that the Merit Release Bird ritual represents a risk for human contamination in countries were the avian influenza virus is circulating.

